# Supplementary material for: Investigation of spatiotemporal evolution and driving mechanisms of carbon sources and sinks in dry-hot valleys under extreme climate events: a case study of the Nu River dry-hot valley
Source: PeerJ. 2025 Nov 3;13:e20268. doi: 10.7717/peerj.20268 (PMC12591053; doi:10.7717/peerj.20268)
Supplement: Supplemental Information 1 [file peerj-13-20268-s001.zip › Supplemental Files/Raw data/Data File Description.docx]

This document provides a detailed description of the three data files supplied: "Extreme climate indicator station data.xlsx", "NPP annual total data.xlsx", and "Pixel extraction to point data.xlsx". The purpose is to ensure clarity for editors, reviewers, and readers regarding the content and structure of these datasets.

1. File: Extreme climate indicator station data.xlsx - Sheet1.csv

General Description:

This file contains observational data from 10 meteorological stations, focusing on various extreme climate indices. It appears to provide annual values for these indices for each station, likely alongside station identification, year, and geographical coordinates. This data is crucial for analyzing trends and patterns in extreme weather and climate events at specific locations.

Description of Common Columns:

Station_ID / Station_Name: Unique identifier or name for each meteorological station.

Year: The year for which the climate index data is recorded.

Longitude: The geographical longitude of the meteorological station.

Latitude: The geographical latitude of the meteorological station.

Meteorological Station Data (10 Stations):

This file includes data for 10 distinct meteorological stations.

| altitude | lon | lat | ID | station |
| --- | --- | --- | --- | --- |
| 1503 | 100.2167 | 23.95 | 569510 | Lincang |
| 2382 | 100.2167 | 26.83333 | 566510 | Lijiang |
| 1649 | 99.18333 | 25.11667 | 567480 | Baoshan |
| 1992 | 100.1833 | 25.7 | 567510 | Dali |
| 2589 | 99.1 | 30 | 562470 | Batang |
| 3320 | 98.88333 | 28.45 | 564440 | Deqing |
| 3307 | 97.16667 | 31.15 | 561370 | Qamdo |
| 1190.9 | 98.8 | 27.07 | 566410 | Fugong |
| 1696.9 | 98.5 | 24.98333 | 567390 | Tengchong |
| 1591.3 | 98.75 | 28.05 | 565330 | Gongshan |

Detailed Description of Extreme Climate Indices (15 Indices):

The following are descriptions for 15 commonly used extreme climate indices, as defined by the Expert Team on Climate Change Detection and Indices (ETCCDI). Please verify that these match the indices present in your data file and their corresponding column headers. The data for these indices are typically presented as annual values per station.

FD (Frost Days):

Definition: Annual count of days when the daily minimum temperature (TN) is less than 0°C.

Units: Days.

Significance: Indicates the frequency of frost occurrence, which can impact agriculture, ecosystems, and infrastructure.

SU (Summer Days):

Definition: Annual count of days when the daily maximum temperature (TX) is greater than 25°C.

Units: Days.

Significance: Represents the occurrence of warm days, relevant for heat stress, energy consumption, and tourism.

ID (Ice Days):

Definition: Annual count of days when the daily maximum temperature (TX) is less than 0°C.

Units: Days.

Significance: Indicates prolonged periods of freezing conditions, affecting transportation, infrastructure, and ecosystems.

TR (Tropical Nights):

Definition: Annual count of days when the daily minimum temperature (TN) is greater than 20°C.

Units: Days.

Significance: Measures the frequency of warm nights, which can impact human health (especially for vulnerable populations) and plant physiology.

GSL (Growing Season Length):

Definition: Annual count of days between the first occurrence of at least 6 consecutive days with daily mean temperature (TG) > 5°C and the first occurrence after July 1st (in the Northern Hemisphere) or January 1st (in the Southern Hemisphere) of at least 6 consecutive days with TG < 5°C.

Units: Days.

Significance: A key indicator for agriculture, representing the period available for plant growth.

TXx (Monthly Maximum Value of Daily Maximum Temperature):

Definition: The highest daily maximum temperature recorded in a given month. If the file contains annual values, this might represent the absolute highest daily maximum temperature of the year, or an annual average of monthly TXx values. Please clarify based on your data processing. For this description, we assume it is the annual maximum of daily maximum temperatures.

Units: °C.

Significance: Indicates extreme heat events during the day.

TNx (Monthly Maximum Value of Daily Minimum Temperature):

Definition: The highest daily minimum temperature recorded in a given month. If the file contains annual values, this could be the annual maximum of daily minimum temperatures (i.e., the warmest night of the year).

Units: °C.

Significance: Reflects periods of unusually warm nights.

TXn (Monthly Minimum Value of Daily Maximum Temperature):

Definition: The lowest daily maximum temperature recorded in a given month. If the file contains annual values, this could be the annual minimum of daily maximum temperatures (i.e., the coolest day of the year).

Units: °C.

Significance: Indicates unusually cool daytime conditions.

TNn (Monthly Minimum Value of Daily Minimum Temperature):

Definition: The lowest daily minimum temperature recorded in a given month. If the file contains annual values, this would be the annual minimum of daily minimum temperatures (i.e., the coldest night of the year).

Units: °C.

Significance: Represents extreme cold events during the night.

DTR (Daily Temperature Range):

Definition: The monthly mean difference between daily maximum temperature (TX) and daily minimum temperature (TN). If the file contains annual values, this would typically be the annual average of these monthly mean differences.

Units: °C.

Significance: Reflects the variability between daytime and nighttime temperatures, which can affect plant growth and human comfort.

Rx1day (Maximum 1-Day Precipitation):

Definition: The highest precipitation amount recorded in a single day within a month (or year, if annually aggregated). If the file contains annual values, this is the annual maximum 1-day precipitation.

Units: mm.

Significance: Indicates the intensity of short-duration heavy rainfall events, relevant for flash floods and erosion.

Rx5day (Maximum 5-Day Precipitation):

Definition: The highest precipitation amount recorded in any consecutive 5-day period within a month (or year, if annually aggregated). If the file contains annual values, this is the annual maximum consecutive 5-day precipitation.

Units: mm.

Significance: Represents prolonged heavy rainfall events, often associated with widespread flooding.

SDII (Simple Daily Intensity Index):

Definition: The annual total precipitation divided by the number of wet days (days with precipitation ≥ 1.0 mm) in the year.

Units: mm/day.

Significance: Measures the average precipitation amount on a wet day, indicating the general intensity of rainfall

R10mm (Number of Heavy Precipitation Days):

Definition: Annual count of days when daily precipitation is greater than or equal to 10 mm.

Units: Days.

Significance: Indicates the frequency of heavy rainfall events.

CDD (Consecutive Dry Days):

Definition: Maximum number of consecutive days in a year where daily precipitation is less than 1 mm.

Units: Days.

Significance: A measure of drought duration, impacting water resources, agriculture, and ecosystems.

2. File: NPP annual total data.xlsx - Sheet1.csv

General Description: This file contains data on the annual total Net Primary Productivity (NPP). NPP is a measure of the net amount of solar energy converted to chemical energy by green plants per unit area per unit time, representing the basis of an ecosystem's food web and carbon cycle.

Description of Columns:

Station_ID / Longitude & Latitude: Identifier for the station or geographical coordinates to link NPP data to specific locations (likely the meteorological stations).

Year: The year for which the NPP data is recorded.

NPP_total / NPP_value: The annual total Net Primary Productivity for the specified location and year.

Units: Typically in grams of carbon per square meter per year (g C/m²/year) or similar units of mass per area per time.

3. File: Pixel extraction to point data.xlsx - Sheet1.csv

General Description: This file likely contains data derived from extracting values from raster (gridded) datasets (such as satellite-derived NPP maps) to specific point locations, which are presumably the meteorological station locations. This process allows for direct comparison and correlation between point-based climate data and spatially continuous data like NPP.

Description of Columns:

Station_ID: Unique identifier for the meteorological station to which the pixel value corresponds.

Year: The year of the data.

Latitude_Station, Longitude_Station: Coordinates of the station.

Latitude_Pixel, Longitude_Pixel: Coordinates of the center of the extracted pixel (if different or for reference).

Extracted_Pixel_Value / NPP_Extracted: The value of the pixel (e.g., NPP) that spatially corresponds to the station's location for a given year.
